# Fecal Microbiota Transplantation Combined with a Low FODMAP Diet for the Treatment of Irritable Bowel Syndrome with Predominant Diarrhea

**DOI:** 10.1155/2022/5121496

**Published:** 2022-09-21

**Authors:** Hong-Li Huang, Jia-Qi Zhu, Liu-Si Yang, Qiong Wu, Di-Wen Shou, Hui-Ting Chen, Jun Ma, Yong-Qiang Li, Hao-Ming Xu, Yong-Jian Zhou

**Affiliations:** ^1^Department of Gastroenterology and Hepatology, The Second Affiliated Hospital, School of Medicine, South China University of Technology, Guangzhou 510180, China; ^2^Department of Gastroenterology and Hepatology, Guangzhou Digestive Disease Center, Guangzhou First People's Hospital, Guangzhou 510180, China; ^3^Department of Traditional Chinese Medicine, Guangzhou First People's Hospital, Guangzhou 510180, China

## Abstract

**Background:**

Fecal microbiota transplantation (FMT) has been found to be effective in irritable bowel syndrome with predominant diarrhea (IBS-D). We conducted this study to determine the impact of a low FODMAP diet (LFD) on the gut microbiota and the efficacy of FMT in the treatment of IBS-D.

**Methods:**

A retrospective analysis of a single-arm open-label prospective study was conducted to investigate the impact of FMT alone (*n* = 40) and FMT+LFD (*n* = 40) in refractory IBS-D. The IBS-quality of life (QOL), IBS-severity scoring system (SSS), gastrointestinal symptom rating scale (GSRS), Hamilton anxiety scale (HAMA), and Hamilton depression scale (HAMD) were used to evaluate the efficacy, and partial 16S rDNA amplicon sequencing was used to profile the microbiota.

**Results:**

The response rates were higher in the FMT+LFD group than in the FMT group (1 mo, 3 mo, 6 mo: 70.0% vs. 55.0%, 67.5% vs. 57.5%, 62.5% vs. 27.5%, respectively). The FMT+LFD group showed significantly better improvement in IBS-QOL at 1, 3, and 6 months; IBS-SSS at 6 months; and GSRS at 1 month compared to FMT alone. Changes in HAMA and HAMD were similar in the two groups. The LFD significantly upregulated the FMT-induced microbial diversity (OTUs: 666 vs. 574, Adonis: *P* = 0.02) and significantly strengthened the upregulation of *Bacteroides*, *Alistipes*, and *Ruminococcaceae_UCG-002* and the downregulation of *Bifidobacterium*.

**Conclusion:**

An LFD enhanced the efficacy of FMT, increased the gut microbial diversity after FMT, and strengthened the inhibitory effect of FMT on conditional pathogens.

## 1. Introduction

Irritable bowel syndrome (IBS) is a functional gastrointestinal disorder characterized by abdominal pain, abdominal bloating, and altered bowel habits. The pathogenesis of IBS includes visceral hypersensitivity, alteration in brain-gut axis, intestinal permeability, gut microbiota, food intolerance, colonic bacterial gas production, and hereditary factors [1]. The global prevalence of IBS is about 11.2% [2]. Patients with IBS have multiple symptoms, but the medical treatment is usually effective in relieving only one or two main symptoms. Moreover, drugs alone are not effective in all cases. Many studies [3] have confirmed that the gut microbiota are affected by dietary habits. In recent years, gut microbiota-targeted therapy [4] and dietary interventions [5] are being increasingly used in clinical practice for the treatment of IBS. The gut microbiota-targeted treatment has been recognized by the Rome guidelines for IBS [6].

Fecal microbiota transplantation (FMT) is an emerging technique of transferring the gut microbiota from healthy individuals to IBS patients in order to obtain new microbial balance and treat their intestinal and extraintestinal diseases. In 1989, McEvoy [7] first used FMT to treat IBS, and the effective rate was 36%. Pinn et al. [8] used FMT to treat patients with refractory IBS, and the remission rate was 70%. The Food and Drug Administration (FDA) has recommended FMT as the treatment for recurrent *Clostridium difficile* infection. FMT has been used to treat IBS in recent years. In a randomized controlled trial involving 165 IBS patients conducted by El-Salhy et al. [9], the response rate was 23.6%, 76.9%, and 89.1% in patients who received placebo (own feces), 30 g FMT, or 60 g FMT, respectively. FMT was associated with significant a change in the gut microbial profile and an improvement in fatigue and the quality of life (QOL) of these patients. However, Halkjaer et al. [10] reported an increase in the diversity of fecal microbiota (FM) of patients with moderate to severe IBS after FMT without significant impact on the clinical symptoms compared to the placebo group. Our previous study [11] found that FMT has obvious short-term benefits in IBS patients, but the long-term effects of FMT were poor. As per the European consensus report on clinical applications and procedures of FMT, FMT is recommended for IBS patients with failure of the standard treatment with continuous disease progression [12].

Recent studies [13, 14] have found that a reduction in the consumption of fermentable oligosaccharides, disaccharides, monosaccharides, and polyols (FODMAP) in IBS patients significantly improves their gastrointestinal symptoms. In 2012, Staudacher et al. [15] proposed for the first time that a low FODMAP diet (LFD) might be effective in relieving the main symptoms of IBS patients. In 2014, Halmos et al. [16] recommended an LFD as the first-line therapy for IBS. A prospective nonrandomized controlled study [17] found that an LFD for 3 weeks significantly reduced abdominal pain and distention with a high rate of patient satisfaction (70.9%). A systematic review and meta-analysis [18] found that an LFD significantly reduced the severity of symptoms; reduced abdominal pain, abdominal distension, and other symptoms; as well as improved the total QOL of patients with IBS. An LFD is considered to act by regulating intestinal microecological balance, maintaining intestinal barrier function, and reducing intestinal inflammatory reaction and abnormal immune response [19]. It reduces the accumulation of intestinal content, reduces the stimulation to highly sensitive viscera, and alleviates the emotional perception related to abdominal distention [20, 21].

Therefore, we designed this study to explore the effect of an LFD combined with FMT on refractory IBS-D and to observe whether the addition of an LFD can increase the efficacy or long-term remission of IBS.

## 2. Materials and Methods

### 2.1. Study Design and Participants

**Figure 1:**
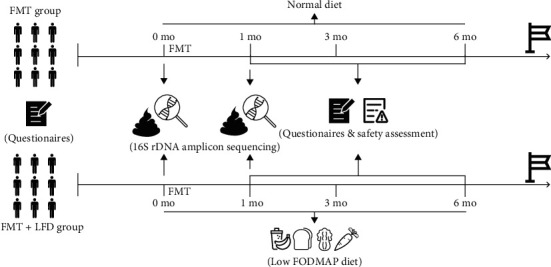
Schematic diagram of the study design.

Patients admitted to the Department of Gastroenterology and Hepatology, Guangzhou First People's Hospital, China, for refractory IBS-D from 2017 to 2021 were prospectively included, and their medical records were retrospectively analyzed. Refractory IBS-D was defined as failure to respond to currently available IBS-D treatment such as dietary changes, antibiotics, probiotics, antidepressants, and psychotherapy that met the Rome IV criteria for IBS-D [22, 23]. Exclusion criteria were as follows: (a) pregnancy or lactation, (b) follow − up duration < 6 months, (c) inability to provide written informed consent, (d) presence of serious systemic diseases, (e) those who did not undergo endoscopic examination, and (f) those who had special dietary habits or were receiving medical dietary intervention. All participants were asked to provide dietary records of one week before enrollment, and our nutritionists decided whether they were suitable for dietary intervention. This study and the FMT protocol were approved by the Medical Ethics Committee of Guangzhou First People's Hospital (no. K-2017-078-02). This study was retrospectively registered on ClinicalTrials.gov (NCT03613545). All the participants provided written informed consent before their enrollment. The patients were divided into two groups according to their willingness to follow an LFD: FMT group and FMT+LFD group. The patients were followed up for 6 months after the treatment. The study design is shown in Figure 1.

Feces donors were carefully selected from middle school or college students using the following protocol [24]: (a) healthy nonpregnant volunteers aged 16-35 years with good dietary habits and healthy lifestyles; (b) no drug use (e.g., antibiotics) within the preceding 6 months; (c) absence of infectious, autoimmune, or other diseases related to gut microbiota or gastrointestinal disorders; and (d) normal laboratory tests including but not limited to complete blood count; erythrocyte sedimentation rate; C-reactive protein; liver and renal function tests; tests for viral hepatitis A, B, and C; human immunodeficiency virus (HIV) and syphilis; and fecal test (stool routine, occult blood test, and infectious pathogen test).

### 2.2. FMT Administration

Fresh feces (150-200 g each) from healthy donors were dissolved in 1,000 ml normal saline. The microbiota were isolated using the GenFMTer automatic purification system (FMT Medical, Nanjing, China), as per the manufacturer's protocol. Using the colonic transendoscopic enteral tube (TET), FM can be directly transported to the terminal ileum, and the number of endoscopic operations can be reduced. We preferentially recommended patients undergo FMT through colonic TET. However, some patients (5/80) who were not willing for FMT through colonic TET underwent FMT by spraying under gastroscope. The two processes were as follows: (1) *colonic TET*: bowel preparation was done using polyethylene glycol-electrolyte solution. Subsequently, a colonoscopy was performed. A TET (FMT Medical, Nanjing, China) was inserted via the anus into the terminal ileum as previously described [25]. The next day post-TET insertion, 150 ml of normal saline containing ~50 cm^3^ centrifuged microbiota was administered into the entire colon via the TET. After FMT, patients were placed in the right lateral position for ≥30 min and were allowed to eat 2 h later. (2) *Gastroscopic spraying*: intramuscular injection of metoclopramide was given 1 h before FMT to prevent vomiting. Intravenous injection of a proton pump inhibitor was given to inhibit gastric acid secretion. Patients were administered 150 ml of normal saline containing ~50 cm^3^ centrifuged microbiota into the second part of the duodenum using a gastroscope. Patients were asked to remain in the half sitting position for at least 4 h after spraying FM. The FMT procedure was repeated every other day for 2-3 times [26].

### 2.3. Dietary Intervention

Patients in the FMT+LFD group received a leaflet containing the information about the LFD of Monash University (Supplementary material Table S1), which includes foods that should be avoided or restricted and foods that should be consumed. The LFD was provided to be taken under the guidance of professional doctors (Department of Nutrition, Guangzhou First People's Hospital). During the follow-up period, the study patients were required to maintain a food diary. Patients in the FMT group continued to eat according to their previous eating habits. The above dietary regimen lasted until the end of the study and the patients were followed up continuously.

The study patients did not receive any antibiotics, probiotics, hormones, or other drugs for at least 3 months pre-FMT and 6 months post-FMT. Their fecal samples were collected before and 1 month after FMT and immediately stored at -80°C in the biological sample bank of our hospital. Their demographic and clinical characteristics were recorded as shown in Table 1.

### 2.4. Assessment of Clinical Efficacy and Safety

All participants were asked to complete standard questionnaires including the IBS-QOL [27, 28], the IBS severity scoring system (IBS-SSS) [29, 30], and the gastrointestinal symptom rating scale (GSRS) [31]. Mental and psychological states were assessed using the Hamilton anxiety scale (HAMA) and Hamilton depression scale (HAMD) [32, 33] before FMT/FMT+LFD, 1 month, 3 months, and 6 months after FMT/FMT+LFD via face-to-face interviews with trained professionals. The responders were defined as patients who achieved improvement in gastrointestinal symptoms (subjective feeling of being better than before treatment) and/or reduction in IBS-QOL (total IBS-QOL score lower than that before treatment) and/or IBS-SSS (at least 1 grade better than before treatment). Otherwise, the patients were defined as nonresponders.

The severity of adverse events was classified into mild (did not affect the daily activities of the subject), moderate (affected the daily activities of the subject to some extent), and severe (significantly affected the daily activities of the subject).

### 2.5. Extraction of Fecal DNA and Analysis of Gut Microbiota

The HiPure Stool DNA Kit (Magen, Guangzhou, China) was used to extract total fecal DNA. The primers used to amplify the 16S rDNA target regions were as follows: 341F: CCTACGGGNGGCWGCAG; 806R: GGACTACHVGGGTATCTAAT [34]. Amplified products of 400-450 bpm were purified by the Phusion High-Fidelity PCR Master Mix (New England Biolabs, Beverly, USA). Sequencing libraries were generated by the TruSeq DNA PCR-Free Sample Preparation Kit (Illumina, San Diego, USA) before adding in index codes. The quality of the constructed library was evaluated by the Agilent Bioanalyzer 2100 system and Qubit@ 2.0 Fluorometer (Thermo Scientific, Carlsbad, USA). The Illumina HiSeq 2500 platform (Novogene Bioinformatics Technology, Tianjin, China) was used for sequencing [34].

### 2.6. Bioinformatics Analyses

#### 2.6.1. Quality Control and Read Assembly

Raw data containing adapters or low-quality reads were filtered as per the FASTP (version 0.18.0) criteria [>10% of unknown nucleotides (N) and <50% of bases with quality (*Q* − value) > 20]. Paired-end clean reads were merged as raw tags using FLSAH (version 1.2.11) with a minimum overlap of 10 bp and mismatch error rates of 2%. Noisy sequences of raw tags were filtered by QIIME (version 1.9.1) under the following filtering conditions: (a) break raw tags from the first low-quality base site where the number of bases in the continuous low-quality value (the default quality threshold was ≤3) reached the set length (the default length was 3), and (b) filter tags with high-quality base length of <75% of the tag length [35].

#### 2.6.2. *α*-Diversity Analysis

QIIME (version 1.9.1) was used to obtain the *α*-diversity indices [36]. The ggplot2 package of the R project (version 2.2.1) was used to perform operational taxonomic unit (OTU) rarefaction and plot rank abundance curves. Tukey's HSD test was used to compare three or more groups. The Shannon index was calculated by Welch's *t*-test in the R project Vegan package (version 2.5.3). In case of two comparative groups with total repeat samples ≥ 3 in each group and the number of species tags/total tags of at least one sample ≥ 0.1%, then the top 1,000 high abundance OTUs were statistically tested with the R language Vegan data package.

#### 2.6.3. OTU Analysis and *β*-Diversity Analysis

Effective tags of OTUs ≥ 97% were clustered together using UPARSE software (version 9.2.64) [37]. Each cluster contained the most abundant tag sequence of each cluster. The multivariate statistical technique of principal coordinate analysis (PCoA) [38] of Bray-Curtis distances was calculated using the Vegan package (version 2.5.3) and plotted using ggplot2 package (version 2.2.1). The analysis of similarity test [39], based on the distance index ranking, was used to determine whether there was a significant difference in the distance between the groups and the distance within the groups in the microbial structure. The Adonis test [40], also known as permutational MANOVA, was used to analyze the interpretation of the groups to the sample difference based on the distance matrix and use the substitution test to analyze the statistical significance of the groups.

#### 2.6.4. Community Composition Analysis

A naive Bayesian model using the RDP classifier (version 2.2) [41] based on SILVA (version 132) [42] or Greengenes (version gg_13_5) [43] databases was used to classify sequences based on organisms. The community composition was visualized by the ggplot2 package (http://CRAN.R-project.org/package=ggplot2) (version 2.2.1) in the R project. The heat map package (version 1.0.12) in R project was used to plot the heat map of genus abundance.

#### 2.6.5. Linear Discriminant Analysis Effect Size Analysis

Linear discriminant analysis (LDA) effect size (LEfSe) analysis (http://huttenhower.sph.harvard.edu/lefse/) was used to identify differences in the taxa between groups. The nonparametric Kruskal-Wallis analysis was used to detect taxa with significant differential abundance. Biological consistency was subsequently investigated using pairwise Wilcoxon rank-sum tests among subclasses. The LDA was used to estimate the effect size of each differentially abundant trait. *α* values of 0.05 were used for the Kruskal-Wallis analysis, and a threshold of 2.0 was chosen for logarithmic LDA scores [44].

#### 2.6.6. Kruskal-Wallis Analysis

The Kruskal-Wallis rank-sum test model was used to identify whether there were differences in the median species abundance among multiple groups. When there were more than two groups for comparison and the number of sample repetitions in the group was more than two, the R language Vegan package was used to carry out the statistical test for the species whose relative abundance (number of species tags/total number of tags) of at least one sample reached more than 0.1% and the high abundance OTUs of the top 1,000 [45].

### 2.7. Statistical Analysis

Data has been presented as numbers, percentages, mean ± standard deviation, or median (quartile 1, quartile 3). Inter- and intragroup differences were analyzed by the Wilcoxon signed rank, unpaired *t*-test, or one-way ANOVA with post hoc Tukey's test as appropriate. The paired Student's *t*-test was used for paired data. The SPSS software (version 23.0; IBM Corp.) was used for all statistical analyses. The *P* values < 0.05 were considered to be significant.

## 3. Results

### 3.1. Characteristics of the Participants

A total of 80 patients with IBS-D were divided into two groups with 40 patients in each. The FMT group was treated with FMT alone, while the FMT+LFD group was treated with FMT combined with an LFD. A total of six healthy donors donated feces (Table 1). The mean age of FMT and FMT+LFD groups was 39.55 years (range 23-67 yr) and 43.2 years (range 20-70 yr), respectively. The mean duration of the disease in the FMT and FMT+LFD groups was 98.60 months (range 8-480 mo) and 108.60 months (range 6-420 mo), respectively. All patients completed the questionnaires at baseline, 1 month, 3 months, and 6 months after FMT. Seventy-five patients underwent FMT by colonic TET, and five patients underwent FMT by gastroscopic spraying. FMT was performed once, twice, and thrice in 1, 6, and 73 patients, respectively.

The response rates of the FMT group at 1 month, 3 months, and 6 months after FMT were 55.0%, 57.5%, and 27.5%, respectively. The response rates of the FMT+LFD group at 1 month, 3 months, and 6 months after FMT were 70.0%, 67.5%, and 62.5%, respectively. The characteristics of the patients and donors are summarized in Table 1.

### 3.2. IBS Symptoms Alleviated after FMT

#### 3.2.1. IBS-QOL

**Figure 2:**
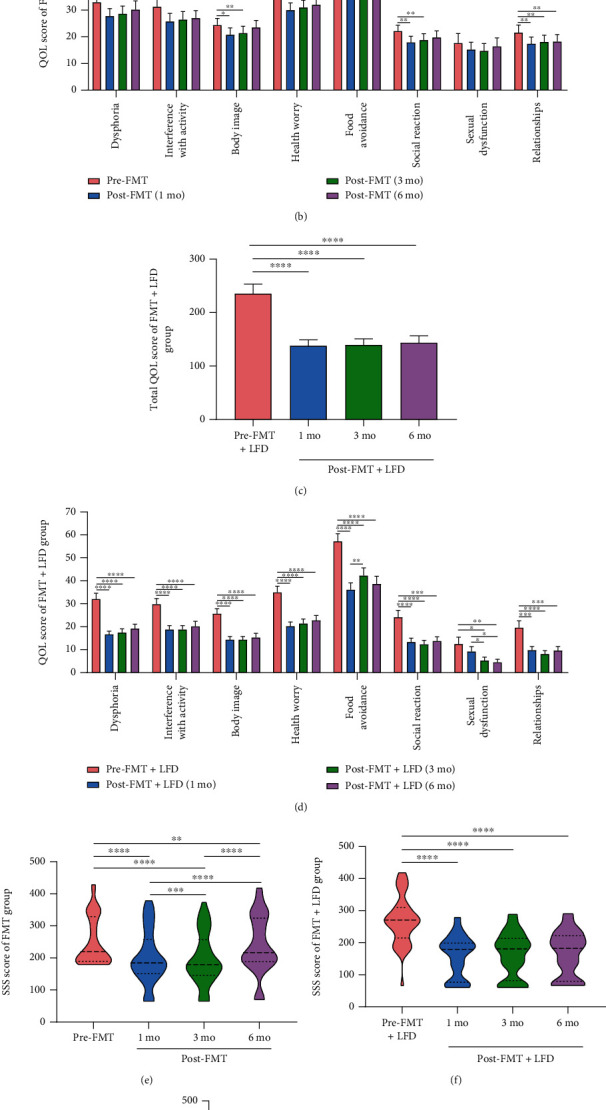
IBS-QOL and IBS-SSS of the FMT and FMT+LFD groups before and after treatment. (a–d) Alterations of total IBS-QOL score (a, c) and 8 dimension scores (b, d) in the FMT group (a, b) and the FMT+LFD group (c, d); (e–g) alterations of IBS-SSS in the FMT group and the FMT+LFD group and comparison between the two groups. ^∗^*P* < 0.05, ^∗∗^*P* < 0.01, ^∗∗∗^*P* < 0.001, ^∗∗∗∗^*P* < 0.0001.

The total IBS-QOL score and the scores of 8 dimensions of the FMT group were calculated as shown in Figures 2(a) and 2(b). In the FMT group, the total IBS-QOL score decreased significantly at 1 month, 3 months, and 6 months after FMT (all *P* < 0.0001) compared to that before FMT. However, there was slight increase in the total IBS-QOL score with time (0 mo, 1 mo, 3 mo, and 6 mo: 242.9 ± 120.6, 198.7 ± 118.6, 203.3 ± 119.5, 211.2 ± 128.4, respectively). At 1 month and 3 months after FMT, except for sexual dysfunction, the other seven dimensions in the FMT group showed significant improvement compared to those before FMT, while only 4 dimensions (interference with activity, health worry, food avoidance, and relationships) showed improvement at 6 months after FMT (Figure 2).

In the FMT+LFD group, as shown in Figures 2(c) and 2(d), the total IBS-QOL score at 1 month, 3 months, and 6 months after FMT and LFD were significantly lower than that before treatment (all *P* < 0.0001). At 1 month, 3 months, and 6 months after FMT and LFD, except for sexual dysfunction (1 mo, 3 mo, and 6 mo: *P* = 0.1133, *P* = 0.0126, and *P* = 0.0068, respectively), the other seven dimensions were significantly reduced compared to those before treatment (Figure 2). In addition, the dimension of food avoidance increased significantly at 3 months after treatment compared to 1 month after treatment (*P* = 0.005).

**Figure 3:**
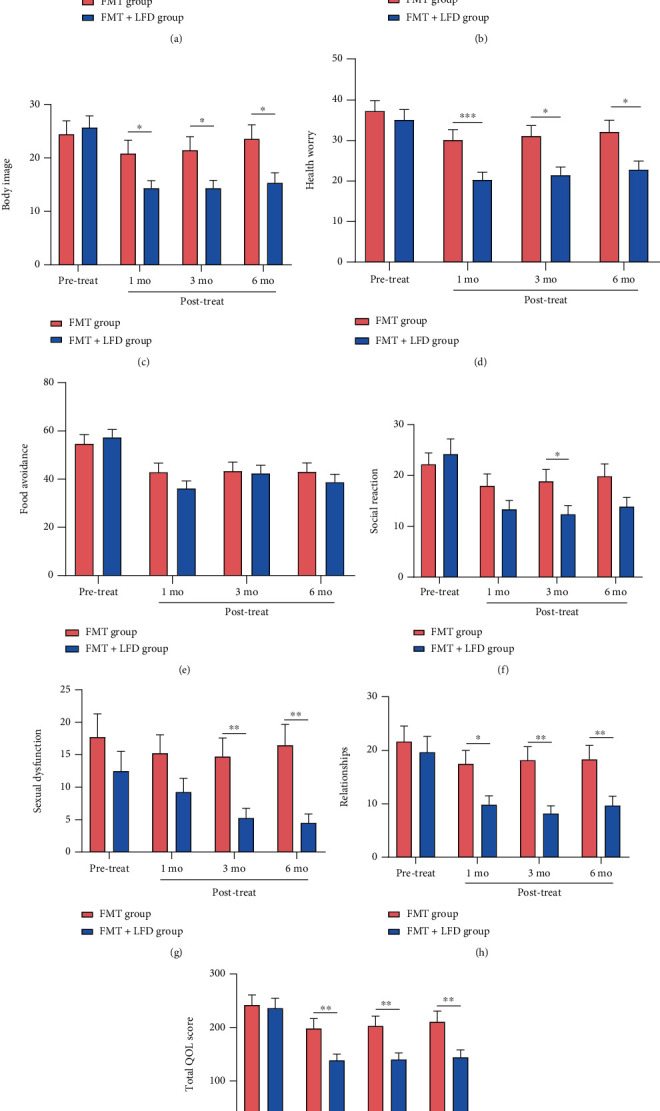
IBS-QOL of the FMT and FMT+LFD groups before and after treatment. (a–h) Comparisons of eight dimensions (a–h: dysphoria, interference with activity, body image, health worry, food avoidance, social reaction, sexual dysfunction, and relationships) between the two groups; (i) comparisons of total IBS-QOL score between the two groups. ^∗^*P* < 0.05, ^∗∗^*P* < 0.01, ^∗∗∗^*P* < 0.001, ^∗∗∗∗^*P* < 0.0001.

The total IBS-QOL score and eight dimensions between the two groups were compared as shown in Figure 3. Apart from food avoidance, there were significant differences in the other seven dimensions and total IBS-QOL scores at 1 month and 3 months after treatment suggesting that FMT+LFD was more effective in improving IBS-QOL than FMT alone. At 6 months after treatment, the FMT+LFD group improved more significantly when compared to the FMT group in terms of dysphoria (*P* = 0.005), body image (*P* = 0.0143), health worry (*P* = 0.016), sexual dysfunction (*P* = 0.001), relationships (*P* = 0.0076), and total IBS-QOL score (*P* = 0.0086).

#### 3.2.2. IBS-SSS

According to the IBS-SSS score, the symptom grade was divided into the following: remission: <75, mild: 75-175, moderate: 176-300, and severe: >300. In the FMT group, there were 11 patients with severe symptoms and 29 patients with moderate symptoms before treatment. One month after FMT, symptoms improved in 4 patients with severe symptoms (3 moderate, 1 mild) and 15 patients with moderate symptoms (11 mild, 4 remission). Three months after FMT, symptoms improved in 5 patients with severe symptoms (4 moderate, 1 mild) and 18 patients with moderate symptoms (14 mild, 4 remission). Six months after FMT, symptom improvement was observed in only 8 patients with moderate severity (6 mild, 2 remission). Combining FMT with an LFD significantly improved the patient's symptom grade. In the FMT+LFD group, there were 11 patients with severe symptoms and 27 patients with moderate symptoms before treatment. One month after FMT, symptoms improved in 11 patients with severe patients (10 moderate, 1 remission) and 15 patients with moderate symptoms (6 mild, 9 remission). Three months after FMT, all 11 patients with severe symptoms continued to have symptom relief (9 moderate, 1 mild, 1 remission), and 15 patients with moderate symptoms showed improvement (6 mild, 9 remission). Six months after FMT, all 11 patients with severe symptoms had symptom relief (8 moderate, 2 mild, 1 remission), and 14 patients with moderate symptoms showed improvement (6 mild, 8 remission).

As shown in Figure 2(e)–2(g), the IBS-SSS of the FMT group significantly decreased at 1 month (*P* < 0.0001) and 3 months (*P* < 0.0001) after FMT [0 mo, 1 mo, and 3 mo: 202.0 (190.0, 330.0), 185.0 (151.3, 258.8), and 180.0 (146.3, 257.5), respectively]. However, the IBS-SSS at 6 months after FMT was higher than that at baseline [0 mo and 6 mo: 202.0 (190.0, 330.0) and 217.5 (190.0, 325.0), respectively; *P* = 0.0044].

Combining FMT with an LFD significantly reduced IBS-SSS (1 mo, 3 mo, and 6 mo: *P* < 0.0001, *P* < 0.0001, and *P* < 0.0001, respectively) in three follow-up points compared to before treatment and maintained stable therapeutic effect. On comparison with the differences between the two groups (Figure 2(g)), FMT combined with an LFD was more effective in improving IBS-SSS than FMT alone at 6 months after treatment (*P* = 0.0003), but no significant difference was observed at 1 month and 3 months (*P* = 0.059 and *P* = 0.1598, respectively).

#### 3.2.3. GSRS

**Figure 4:**
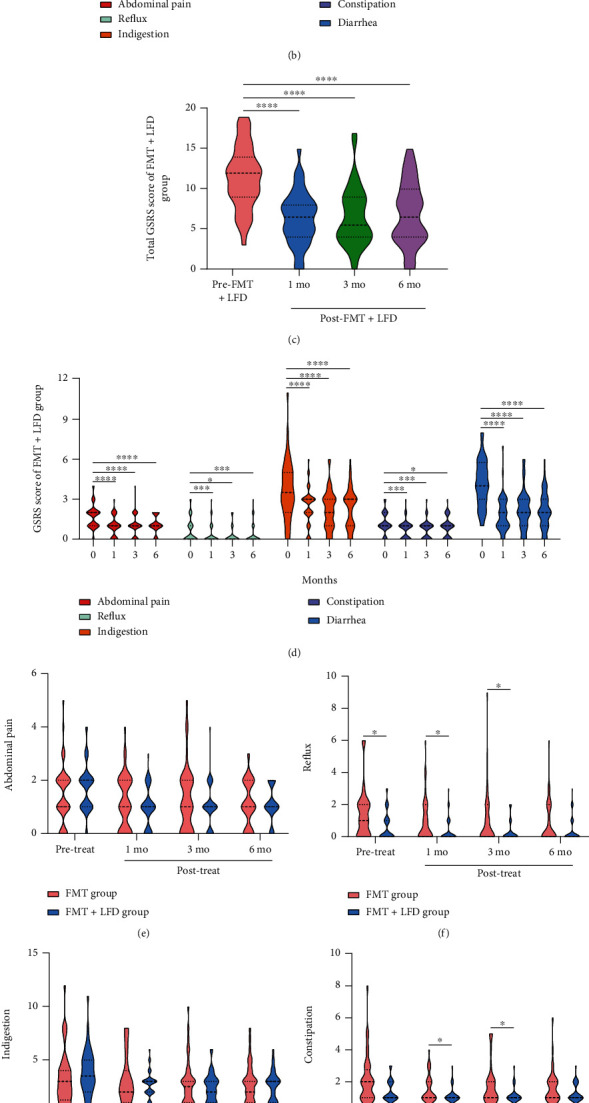
GSRS of the FMT and FMT+LFD groups before and after treatment. (a–d) Alterations of total GSRS score (a, c) and 5 symptoms (b, d) in the FMT group and the FMT+LFD group; (e–i) comparisons of 5 symptoms (e–i: abdominal pain, reflux, indigestion, constipation, and diarrhea) between the two groups; (j) comparisons of total GSRS between the two groups. ^∗^*P* < 0.05, ^∗∗^*P* < 0.01, ^∗∗∗^*P* < 0.001, ^∗∗∗∗^*P* < 0.0001.

The total GSRS and the scores of five symptoms of the FMT group were calculated as shown in Figures 4(a) and 4(b). The total GSRS significantly decreased at 1 month, 3 months, and 6 months after FMT (*P* < 0.0001). At 1 month after FMT, except for indigestion, the other four symptoms in the FMT group significantly improved (*P* = 0.0346, *P* = 0.0035, *P* = 0.0009, *P* < 0.0001). At 3 months and 6 months after FMT, except for abdominal pain, the other 4 symptoms in the FMT group showed significant improvement. In the FMT+LFD group, as shown in Figures 4(c) and 4(d), the total GSRS at 1 month, 3 months, and 6 months after FMT+LFD was significantly lower than that before treatment (all *P* < 0.0001). At all follow-up points after FMT+LFD, all symptoms were significantly improved compared to before treatment.

On comparison of total GSRS and the five symptoms between the two groups (Figures 4(e)–4(j)), there was no significant difference between FMT and FMT+LFD in three dimensions (abdominal pain, indigestion, and diarrhea). Combining FMT with an LFD was more effective in improving reflux (1 mo vs. 0 mo and 3 mo vs. 0 mo: *P* = 0.0382 and *P* = 0.0191, respectively) and constipation (1 mo vs. 0 mo and 3 mo vs. 0 mo: *P* = 0.0317 and *P* = 0.0270, respectively), but unfortunately, reflux was statistically different before FMT (*P* = 0.0102), so we could not judge whether it was caused by the difference of baseline data. However, FMT combined with LFD was more effective than FMT alone in improving total GSRS at 1 month (*P* = 0.0399) but had similar improvement at 3 months (*P* = 0.0824) and 6 months after treatment (*P* = 0.4436).

#### 3.2.4. HAMA and HAMD

HAMA score higher than 7 (of 56) indicated the presence of anxiety symptoms. In the FMT group, there were 23 patients with anxiety symptoms before treatment. At one and three months after FMT, patients with anxiety symptoms reduced to 19. At 6 months after FMT, patients with anxiety symptoms reduced to 15. In the FMT+LFD group, there were 22 patients who had anxiety symptoms before treatment. At one and three months after FMT, patients with anxiety symptoms reduced to 12. Six months after FMT, there were 15 patients with anxiety symptoms.

**Figure 5:**
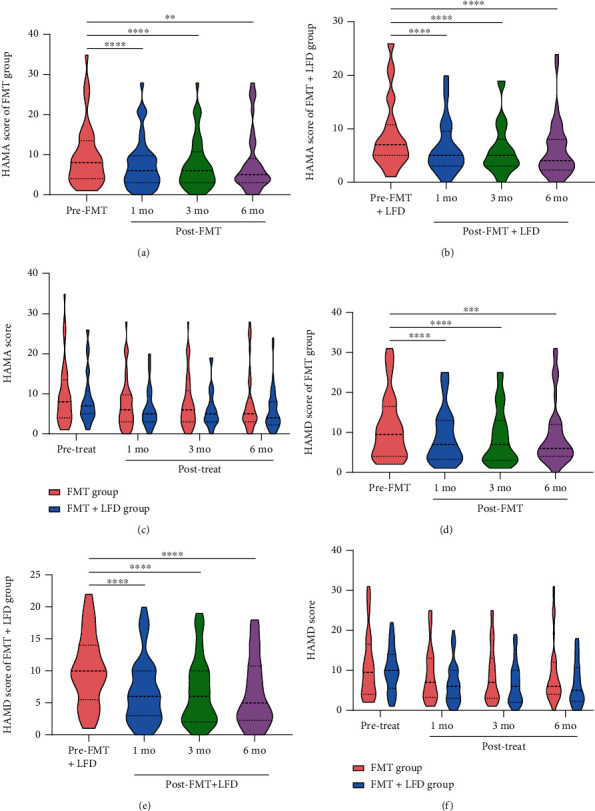
Changes in HAMA and HAMD scores of the FMT group (a, d) and FMT+LFD group (b, e) and their comparison (c, f). (a, b) Alterations of HAMA score in the FMT group and the FMT+LFD group; (c) comparisons of HAMA score between the two groups; (d, e) alterations of HAMD score in the FMT group and the FMT+LFD group; (f) comparisons of HAMD score between the two groups. ^∗∗^*P* < 0.01, ^∗∗∗^*P* < 0.001, ^∗∗∗∗^*P* < 0.0001.

As shown in Figures 5(a)–5(c), the HAMA score of both the FMT group and the FMT+LFD group decreased significantly at 1 month (*P* < 0.0001 and *P* < 0.0001, respectively), 3 months (*P* < 0.0001 and *P* < 0.0001, respectively), and 6 months (*P* = 0.0040 and *P* = 0.0002, respectively) after treatment. The differences between the two groups at the three follow-up points were insignificant (1 mo, 3 mo, and 6 mo: *P* = 0.5464, *P* = 0.4343, and *P* = 0.4228, respectively).

Similarly, HAMD score higher than 8 (of 76) indicated the presence of depressive symptoms. In the FMT group, 24 patients had depressive symptoms before treatment. At one and six months after FMT, patients with depressive symptoms reduced to 18. At 6 months after FMT, patients with depressive symptoms reduced to 16. In the FMT+LFD group, there were 27 patients with depressive symptoms before treatment. At 1, 3, and 6 months after FMT, patients with depressive symptoms reduced to 14, 15, and 15, respectively.

As shown in Figures 5(d)–5(f), the HAMD score of both the FMT group and the FMT+LFD group decreased significantly at 1 month (*P* < 0.0001 and *P* < 0.0001, respectively), 3 months (*P* < 0.0001 and *P* < 0.0001, respectively), and 6 months (*P* = 0.0010 and *P* < 0.0001, respectively) after treatment. The HAMD scores of the two groups were similar at the three follow-up points (1 mo, 3 mo, and 6 mo: *P* = 0.2338, *P* = 0.2203, and *P* = 0.0971, respectively).

### 3.3. Safety of FMT

Adverse events were noted in four patients (5%) (4/80). Two patients reported an increased frequency of passing stools within 12 h after FMT (one in each group). Two patients reported mild abdominal distension and increased passage of flatus within 12 h after FMT (one in each group). However, all symptoms disappeared without medical intervention within 24 h.

### 3.4. Alterations of Diversity and Dominant Microbiota

**Figure 6:**
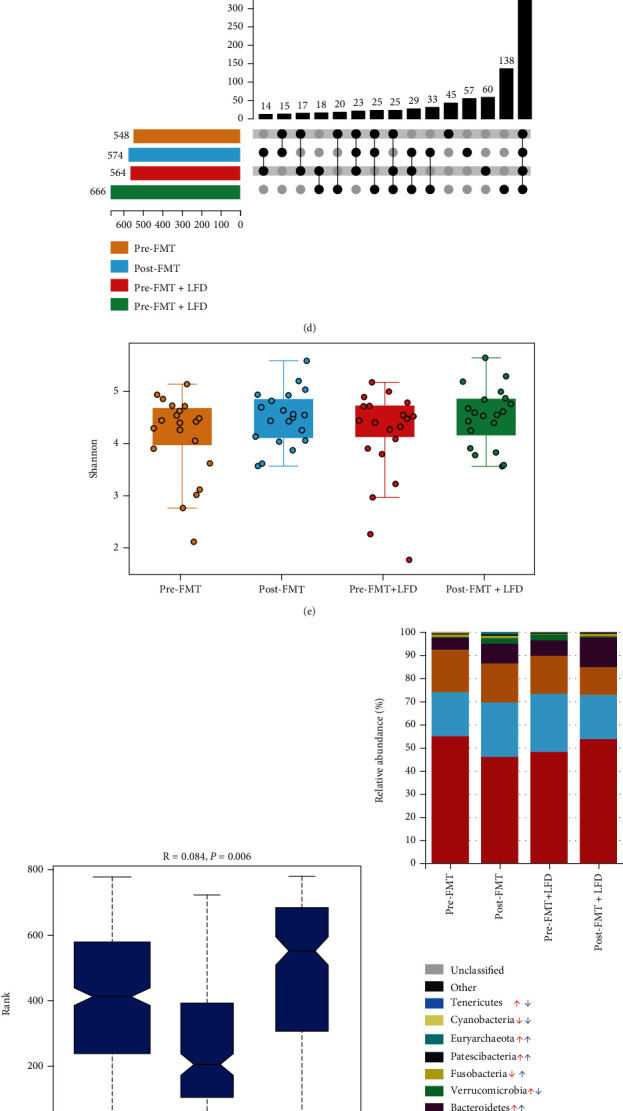
Changes in the dominant microbiota after treatment in the two groups. (a–c) The number of OTUs in IBS patients increased after treatment (a), *α*-diversity increased (b), and the microbial structure changed (c); (d–f) FMT combined with an LFD increased the number of OTUs (d) and *α*-diversity (e) and also lead to significant differences of *β* diversity; (g–i) FMT combined with LFD and FMT alone mediated different dominant microbiota changes in IBS-D patients at the phylum (g), family (h), and genus (i) levels. Upset plot: the horizontal column on the left side of the figure shows the number of OTUs; the lower right is the dot matrix of the intersection part. A single node represents the OTUs unique to the corresponding group, and the connection of multiple points represents the OTUs shared between groups; The upper right is the number of shared/unique OTUs represented by the corresponding lattice. Shannon index: the abscissa represents the grouping (expressed in different colors), and the ordinate represents the size of the Shannon index. The maximum value: the upper horizontal line, the minimum value: the lower horizontal line, the median: the middle line of the box, the upper quartile: the top edge of the box, and the lower quartile: the bottom edge of the box. PCoA: the closer the center distance of each group is, the more similar the microbial structure is. The greater the sum of PCoA1+PCoA2, the greater the difference of microbial structure between groups (*β* diversity). Diversity difference analysis between groups: the vertical axis of the box chart represents the distance ranking, the horizontal axis between represents the distance between groups, and others represent the distance within the corresponding group; The *R* value indicates the degree of difference between groups and within groups, ranging from -1 to 1. The closer the *R* value is to 1, the greater the distance is between groups compared to that within groups; *P* value indicates the significant difference between groups and within groups. Relative abundance stacking diagram: select the top ten phylum/family with relative abundance to draw the stacking diagram, and one color represents one phylum/family. Relative abundance clustering heat map: each row represents a genus, each column represents a group, and color represents genus abundance. The closer the color is to dark blue, the lower the abundance is, and the closer it is to red, the higher the abundance is. The legend shows the abundance value of the corresponding species after normalization. Red arrows indicate Pre-FMT vs. Post-FMT, blue arrows indicate Pre-FMT+LFD vs. Post-FMT+LFD, upward arrows indicate increase, and downward arrows indicate decrease. ^∗^*P* < 0.05, ^∗∗^*P* < 0.01.

**Figure 7:**
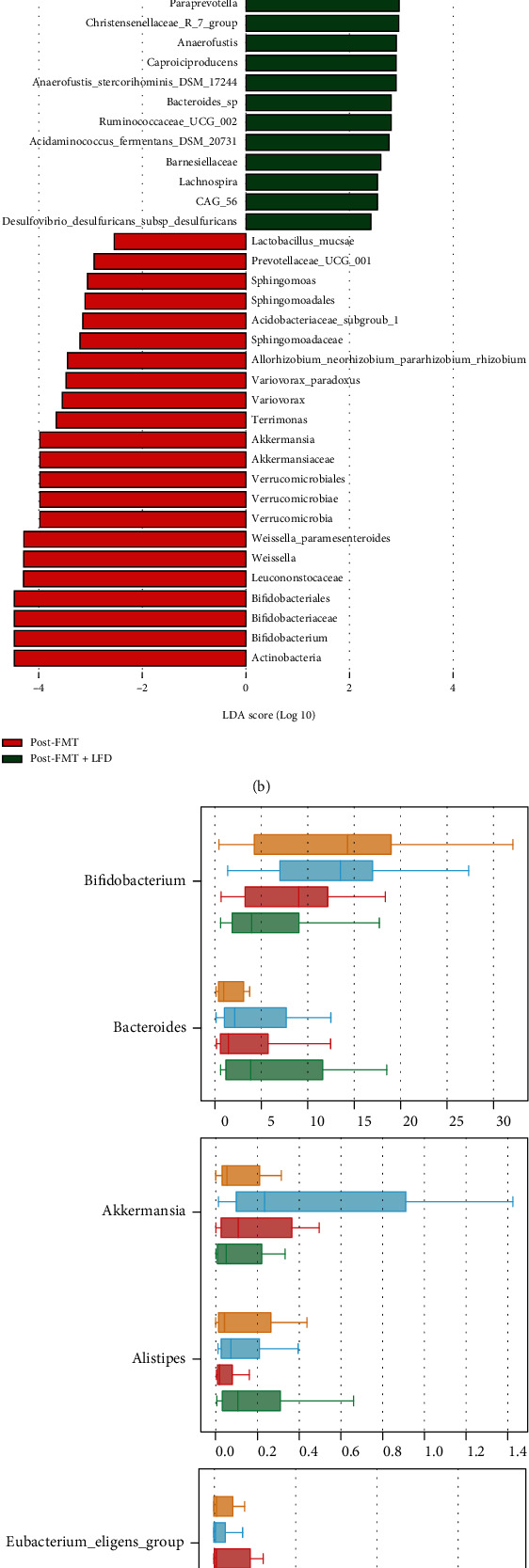
Comparison of the microbiota of FMT+LFD and FMT groups. (a, b) LEfSe analysis showed that the microbiota of the FMT+LFD group was different to that of the FMT group. (a) LDA score chart shows the biomarker of different groups, and the length of the histogram represents the impact of different species (i.e., LDA score). The results with LDA ≥ 2 are retained. (b) Evolutionary branch diagram, the circle radiating from inside to outside represents the classification level from phylum to species, which is displayed to species by default. Each small circle at different classification levels represents a species under the classification level, and the diameter of the small circle is directly proportional to the relative abundance. (c) Kruskal-Wallis analysis showed that the relative abundance of various bacteria was different in the two groups before and after treatment.

Patients were required to collect fecal samples in a sterile sampling tube and directly transfer to -80°C in the hospital, which increased the difficulty of sample collection. A total of 40 samples from 40 patients (FMT group = 20, FMT + LFD group = 20) were provided for 16S rDNA amplicon sequencing. In these 40 patients, FM from healthy donors was collected before FMT (if the FMT group and the FMT+LFD group patients share the same FM, only one sample was tested). Finally, 58 FM samples from healthy donors were included for 16S rDNA amplicon sequencing. The results are shown in Figures 6 and 7.

As shown in Figures 6(a)–6(c), the number of OTUs of healthy donors, patients before treatment, and patients after treatment was 499, 577, and 643, respectively (since there were only 6 donors, the number of OTUs in the donor group is relatively small). The Shannon index of the donor group was significantly higher than that of patients before treatment (*P* = 0.0075). The Shannon index in patients after treatment was significantly higher than that before treatment (*P* = 0.0175). Using PCoA, it was seen that the spatial distance between the donor and patient groups was the farthest. After treatment, the microbial structure of patients came close to the donor group. In order to observe the impact of an LFD on the microbial changes, we divided the samples into four groups: pre-FMT group (before FMT alone), post-FMT group (after FMT alone), pre-FMT+LFD group (before FMT combined with an LFD), and post-FMT+LFD group (after FMT combined with an LFD). As shown in Figures 6(d)–6(f), patients in the FMT+LFD group (666 vs. 564) had a higher increase in the OTUs compared to those in the FMT group (574 vs. 548). The Shannon indexes of the two groups were similar (*P* = 0.0924, *P* = 0.1033). In order to observe whether the two treatment methods mediated the difference in microbiota, we used Anosim (*P* = 0.0060) and Adonis (*P* = 0.0200) tests which showed significant alterations.

For analysis of dominant microbiota, we analyzed the top ten microbiota in each group based on OTUs and drew the stacking diagram of relative abundance at the phylum level (Figure 6(g)) and family level (Figure 6(h)) and the clustering heat map of the top twenty microbiota at the genus level (Figure 6(i)). In total, FMT alone upregulated *Weissella*, *Bacteroides*, *Escherichia-Shigella*, *Akkermansia*, *Enterococcus*, *Parabacteroides*, *Collinsella*, *Actinomyces*, *Eubacterium_hallii*, and *Dorea* and downregulated *Streptococcus*, *Lactobacillus*, *Romboutsia*, *Bifidobacterium*, *Erysipelotrichaceae_UCG-003*, *Subdoligranulum*, *Pediococcus*, *Blautia*, *Faecalibacterium*, and *Fusobacterium*; FMT combined with LFD upregulated *Bacteroides*, *Pediococcus*, *Lactobacillus*, *Parabacteroides*, *Romboutsia*, *Fusobacterium*, *Faecalibacterium*, *Collinsella*, and *Enterococcus* and downregulated *Bifidobacterium*, *Streptococcus*, *Escherichia-Shigella*, *Akkermansia*, *Blautia*, *Subdoligranulum*, *Eubacterium_hallii*, *Erysipelotrichaceae_UCG-003*, *Weissella*, *Actinomyces*, and *Dorea*. The mean relative abundance and *P* values of the groups at all the three levels are shown in Supplementary material Table S2.

### 3.5. Changes of Microbial Composition

To find the difference in the microbiota between the FMT+LFD and FMT groups in IBS-D, we used the Kruskal-Wallis analysis method and found six prominent bacteria at the genus level (Figure 7(c)): *Bifidobacterium*, *Bacteroides*, *Akkermansia*, *Alistipes*, *Eubacterium_eligens_group*, and *Ruminococcaceae_UCG-002*. It was found that the combination of an LFD could strengthen the upregulation of *Bacteroides*, *Alistipes*, and *Ruminococcaceae_UCG-002* and the downregulation of *Bifidobacterium*, but the effect was opposite on *Akkermansia* and *Eubacterium_eligens_group*. Alone, FMT significantly increased the abundance of *Akkermansia*, while the combination with LFD significantly decreased it (Supplementary material Table S3).

LSfSe analysis was used to compare the different microbiota between the two groups. The results are shown in Figures 7(a) and 7(b). Compared with the FMT+LFD group, FMT alone enriched the following: *Bifidobacterium* (LDA = 4.4734, *P* = 0.0186), *Weissella* (LDA = 4.2907, *P* = 0.0373), *Weissella_paramesenteroides* (LDA = 4.2864, *P* = 0.0425), *Akkermansia* (LDA = 3.9772, *P* = 0.0068), *Terrimonas* (LDA = 3.6652, *P* = 0.0377), *Variovorax* (LDA = 3.5474, *P* = 0.0377), *Variovorax_paradoxus* (LDA = 3.4725, *P* = 0.0377), *Allorhizobium_Neorhizobium_Pararhizobium_Rhizobium* (LDA = 3.4451, *P* = 0.0377), *Sphingomonas* (LDA = 3.0591, *P* = 0.0324), *Prevotellaceae_UCG_001* (LDA = 2.9311, *P* = 0.0274), and *Lactobacillus_mucosae* (LDA = 2.5439, *P* = 0.0280), whereas the FMT+LFD group enriched the following: *Pediococcus* (LDA = 4.1446, *P* = 0.0398), *Pediococcus_pentosaceus* (LDA = 4.1424, *P* = 0.0398), *Pseudomonas_veronii* (LDA = 3.4164, *P* = 0.0081), *Eubacterium_eligens_group* (LDA = 3.1982, *P* = 0.0040), *Parabacteroides_goldsteinii* (LDA = 3.1771, *P* = 0.0372), *Eubacterium_oxidoreducens_group* (LDA = 3.1282, *P* = 0.0155), *Paraprevotella* (LDA = 2.9595, *P* = 0.0304), *Christensenellaceae_R_7_group* (LDA = 2.9484, *P* = 0.0483), *Anaerofustis* (LDA = 2.9035, *P* = 0.0212), *Caproiciproducens* (LDA = 2.9018, *P* = 0.0419), *Anaerofustis_stercorihominis_DSM_17244* (LDA = 2.8997, *P* = 0.0212), *Bacteroides_sp* (LDA = 2.8048, *P* = 0.0004), *Ruminococcaceae_UCG_002* (LDA = 2.8038, *P* = 0.0200), *Acidaminococcus_fermentans_DSM_20731* (LDA = 2.7634, *P* = 0.0090), *Lachnospira* (LDA = 2.5386, *P* = 0.0424), *CAG_56* (LDA = 2.5386, *P* = 0.0453), and *Desulfovibrio_desulfuricans_subsp_desulfuricans* (LDA = 2.4151, *P* = 0.0229) (Supplementary material Table S4).

## 4. Discussion

In the current study of 80 patients with refractory IBS-D, we found that the response rates of the FMT group at 1 month, 3 months, and 6 months were 55.0%, 57.5%, and 27.5%, respectively, while those of the FMT+LFD group were 70.0%, 67.5%, and 62.5%, respectively, suggesting that adding an LFD to FMT increased its response rate and long-term remission. The IBS-QOL showed that both treatments effectively improved the patients' QOL. Single FMT treatment was effective in seven dimensions at 1 month except sexual dysfunction, while it was only effective in the following four dimensions at 6 months after FMT: interference with activity, health worry, food avoidance, and relationship. Adding an LFD not only made FMT effective in all dimensions but also resulted in better outcomes at 6 months. However, we also found that the food avoidance of patients increased significantly at 3 months compared to one month after treatment in the FMT+LFD group. It might be because some patients were tired of consuming an LFD after one month. However, after consultation with our nutritionist, the patients in the LFD group could appropriately adjust their diet and maintain good compliance. In addition, we noticed a significant improvement in sexual function of the patients in the FMT+LFD group, which may be related to an LFD, but the exact reasons could not be determined. On comparing the total IBS-QOL score and eight dimensions between the two groups, we found that FMT combined with an LFD had a significantly better effect than FMT alone on IBS-SSS and all dimensions except food avoidance. The FMT with and without an LFD showed significant improvement in IBS-SSS, but the efficacy gradually waned off at six months after treatment in the FMT alone group while the effect persisted in the FMT+LFD group. The GSRS showed that FMT in combination with an LFD had better improvement in abdominal pain, reflux, and dyspepsia. A previous study reported that an LFD could effectively alleviate the symptoms of abdominal distension and abdominal pain by reducing intestinal transport load (water and gas) and stimulation [46]. The FMT also improved HAMA and HAMD scores, although the addition of LFD did not strongly affected these scores.

We sequenced 16S rDNA amplicons from fecal samples of 40 IBS-D patients and found that both treatments increased the patients' microbial diversity, as reflected by an increase of OTUs and the Shannon index. By observing the changes of dominant microbial structure at the phylum, family, and genus levels, we found that the increase of *Bacteroidetes* (phylum), *Bacteroidaceae* (family), *Bacteroides* (genus), and *Parabacteroides* (genus) and the decrease of *Actinobacteria* (phylum), *Lachnospiraceae* (family), *Bifidobacteriaceae* (family), *Subdoligranulum* (genus), *Blautia* (genus), and *Bifidobacterium* (genus) was more obvious with FMT+LFD. However, the effect of FMT+LFD on *Firmicutes* (phylum), *Verrucomicrobia* (phylum), *Proteobacteria* (phylum), *Lactobacillaceae* (family), *Ruminococcaceae* (family), *Peptostreptococcaceae* (family), *Enterobacteriaceae* (family), *Pediococcus* (genus), *Lactobacillus* (genus), *Romboutsia* (genus), *Faecalibacterium* (genus), *Akkermansia* (genus), and *Escherichia-Shigella* (genus) was opposite to that observed with FMT alone. By further analyzing the different genus/species, we found that an LFD strengthened the FMT-induced upregulation of *Bacteroides*, *Alistipes*, and *Ruminococcaceae_UCG-002* and the FMT-induced downregulation on *Bifidobacterium*, but the effect was opposite on *Akkermansia* and *Eubacterium_eligens_group*. These findings were similar to those reported by previous studies [47, 48]. Studies have found that an LFD was not conducive for the proliferation of short chain fatty acids (SCFAs) producing bacteria (*Lachnospiraceae*, *Bifidobacteriaceae*, *Lactobacillaceae*, *Ruminococcaceae*, *Faecalibacterium*, *Akkermansia*, *Blautia*, and *Bifidobacterium)* as they require a high FODMAP diet as the substrate to produce SCFAs. The SCFAs can regulate intestinal pH value and prevent abnormal cell proliferation [49] and can induce IBS symptoms. Previous studies have found that the SCFA family induces visceral hypersensitivity in mice, damages intestinal barrier function, and causes chronic mucosal inflammation and abdominal distension [50, 51]. At the same time, SCFAs can activate intestinal and autonomic nerves, leading to changes in the intestinal motility and secretion and cause reflux and abdominal distention [52, 53]. At present, studies have confirmed that FMT (especially in inflammatory bowel disease) can significantly increase the relative abundance of SCFA-producing bacteria and mediate the increase of intestinal SCFAs (especially butyric acid) [54, 55]. However, the concentration of SCFAs in feces was not directly measured in the present study. Hence, the changes in the concentration of SCFAs could be indirectly judged by the relative abundance of SCFA-producing bacteria. Excessive SCFAs may be a double-edged sword for IBS patients, so the combination of an LFD may play a balanced role. These findings suggest that the addition of an LFD to FMT was effective in alleviating the IBS symptoms by increasing microbial diversity, balancing the abundance of SCFA-producing bacteria, and increasing the inhibitory effect of FMT on conditional pathogens (e.g., *Escherichia-Shigella*).

In addition, some epidemiological studies have shown that IBS is more frequent in females than males, while some Asian and Chinese studies have suggested that there is no significant difference between them with IBS-D being more common in males [56–58]. In our study, there were more males than females (52 vs. 28). Higher percentage of male IBS patients in this study may be the reflection of IBS population in southern China. Also, male patients with refractory IBS might be more acceptable to medical therapies especially FMT (which is considered a little “disgusting” and requires certain psychological preparation). Furthermore, this study has some limitations which should be taken into consideration while interpreting the results. First, we could not explore the effect of an LFD alone on gut microbiota and symptoms of patients with refractory IBS-D because most of the study patients had come to our hospital for FMT. Hence, it was difficult to obtain consent from patients for an LFD alone. Second, it was not a randomized controlled trial study, and the influence of the placebo effect on this study could not be excluded. A meta-analysis [59] found that the remission rate of FMT in IBS was close to 60.0%, which was similar to that of the placebo group. Hence, single-arm studies can overestimate the efficacy of any intervention in IBS. Third, although the LFD was planned and supervised by a nutritionist in the study, we still could not rule out the impact of personal dietary preferences on symptoms and gut microbiota. The microbial analysis found that the intragroup difference in the FMT combined with LFD group was greater than the FMT group, which may be caused by less rigorous/single dietary intervention. Although we used feces from strictly screened healthy donors as controls, healthy persons can also have IBS, so the gut microbial structure may overlap between them. Fourth, IBS is a functional disorder. This study mainly included the commonly used scales (e.g., IBS-QOL) for determining the efficacy of FMT and LFD. Although the change in gut microbiota was a relatively objective index, there were still many influencing factors. Moreover, because many study patients had come from far-away places, the change in microbiota at 3 and 6 months after treatment could not be carried out. Future prospective randomized controlled trials with larger sample sizes and longer follow-ups are required to validate the findings of this study.

## 5. Conclusions

This study found that an LFD enhances the efficacy of FMT as reflected by the IBS-QOL, IBS-SSS, and GSRS scores; increases the microbial diversity after FMT; reduces the excessive growth of SCFA-producing bacteria to a certain extent; and strengthens the inhibitory effect of FMT on conditional pathogens.

## Figures and Tables

**Table 1:** Baseline information of patients with IBS-D and donors.

	FMT group	FMT+LFD group	*P* value
Number of patients	40	40	—
Sex (female/male)	12/28	16/24	0.3484
Age (years) (min, max)	39.55 (23 yr, 67 yr)	43.20 (20 yr, 70 yr)	0.2103
Disease duration (min, max)	98.60 (8 mo, 480 mo)	108.60 (6 mo, 420 mo)	0.6439
Delivery			0.6442
Colonic TET	37	38	—
Gastroscope spraying	3	2	—
FMT times			0.4316
3	37	36	—
2	2	4	—
1	1	0	—
Response rate			0.3133
1 mo	55.0% (22/40)	70.0% (28/40)	—
3 mo	57.5% (23/40)	67.5% (27/40)	—
6 mo	27.5% (11/40)	62.5% (25/40)	—
Number of donations			0.2509
Donor no.1 (male, 35)	11	11	—
Donor no.2 (male, 16)	1	1	—
Donor no.3 (male, 28)	47	62	—
Donor no.4 (male, 25)	10	12	—
Donor no.5 (male, 26)	46	30	—
Donor no.6 (female, 25)	1	0	—

FMT: fecal microbiota transplantation; LFD: low FODMAP diet; TET: transendoscopic enteral tubing.

## Data Availability

The original contributions presented in the study are publicly available. These data can be found here: https://submit.ncbi.nlm.nih.gov/subs/sra/SUB10907576/overview, BioProject ID is PRJNA 795467. Other datasets used and analyzed during the current study are available from the corresponding authors on reasonable request.

## Abbreviations

IBS: Irritable bowel syndrome
IBS-D: Irritable bowel syndrome with predominant diarrhea
IBS-C: Irritable bowel syndrome with predominant constipation
IBS-M: Irritable bowel syndrome with mixed bowel habits
IBS-U: Irritable bowel syndrome unclassified
FMT: Fecal microbiota transplantation
FM: Fecal microbiota
FODMAP: Fermentable oligosaccharides, disaccharides, monosaccharides, and polyols
LFD: Low fermentable oligosaccharide, disaccharide, monosaccharide, and polyol diet
SCFAs: Short chain fatty acids
RCT: Randomized controlled trial
IBS-QOL: Irritable bowel syndrome quality of life
IBS-SSS: Irritable bowel syndrome symptom severity scale
GSRS: Gastrointestinal symptom rating scale
HAMA: Hamilton anxiety
HAMD: Hamilton depression
TET: Transendoscopic enteral tubing
OTUs: Operational taxonomic unit
PCoA: Principal coordinate analysis
LDA: Linear discriminant analysis
LEfSe: Linear discriminant analysis effect size.

## Supplementary Materials

Supplementary Materials Table S1: Common low FODMAP and high FODMAP foods. 2, Table S2: The mean relative abundance and P values of each group at all the three levels. 3, Table S3: Relative abundance of genus and Kruskal-Wallis analysis. 4, Table S4: LEfSe analysis between two groups.

## References

[1] Videlock E. J., Chang L. (2021). Latest insights on the pathogenesis of irritable bowel syndrome. *Gastroenterology Clinics of North America*.

[2] Canavan C., West J., Card T. (2014). The epidemiology of irritable bowel syndrome. *Clinical Epidemiology*.

[3] Rinninella E., Cintoni M., Raoul P. (2019). Food components and dietary habits: keys for a healthy gut microbiota composition. *Nutrients*.

[4] Singh P., Alm E. J., Kelley J. M. (2022). Effect of antibiotic pretreatment on bacterial engraftment after fecal microbiota transplant (FMT) in IBS-D. *Gut Microbes*.

[5] Chumpitazi B. P., Hollister E. B., Oezguen N. (2014). Gut microbiota influences low fermentable substrate diet efficacy in children with irritable bowel syndrome. *Gut Microbes*.

[6] Simrén M., Barbara G., Flint H. J. (2013). Intestinal microbiota in functional bowel disorders: a Rome foundation report. *Gut*.

[7] McEvoy R. (1989). Bowel-flora alteration: a potential cure for inflammatory bowel disease and irritable bowel syndrome?. *The Medical Journal of Australia*.

[8] Pinn D., Aroniadis O., Brandt L. J. (2013). Follow-up study of fecal microbiota transplantation (FMT) for the treatment of refractory irritable bowel syndrome (IBS). *The American Journal of Gastroenterology*.

[9] El-Salhy M., Hatlebakk J. G., Gilja O. H., Brathen Kristoffersen A., Hausken T. (2020). Efficacy of faecal microbiota transplantation for patients with irritable bowel syndrome in a randomised, double-blind, placebo-controlled study. *Gut*.

[10] Halkjaer S. I., Christensen A. H., Lo B. Z. S. (2018). Faecal microbiota transplantation alters gut microbiota in patients with irritable bowel syndrome: results from a randomised, double-blind placebo-controlled study. *Gut*.

[11] Huang H. L., Chen H. T., Luo Q. L. (2019). Relief of irritable bowel syndrome by fecal microbiota transplantation is associated with changes in diversity and composition of the gut microbiota. *Journal of Digestive Diseases*.

[12] Konig J., Siebenhaar A., Hogenauer C. (2017). Consensus report: faecal microbiota transfer - clinical applications and procedures. *Alimentary Pharmacology & Therapeutics*.

[13] Chumpitazi B. P., Cope J. L., Hollister E. B. (2015). Randomised clinical trial: gut microbiome biomarkers are associated with clinical response to a low FODMAP diet in children with the irritable bowel syndrome. *Alimentary Pharmacology & Therapeutics*.

[14] Naseri K., Dabiri H., Rostami-Nejad M. (2021). Influence of low FODMAP-gluten free diet on gut microbiota alterations and symptom severity in Iranian patients with irritable bowel syndrome. *BMC Gastroenterology*.

[15] Staudacher H. M., Lomer M. C., Anderson J. L. (2012). Fermentable carbohydrate restriction reduces luminal bifidobacteria and gastrointestinal symptoms in patients with irritable bowel syndrome. *The Journal of Nutrition*.

[16] Halmos E. P., Power V. A., Shepherd S. J., Gibson P. R., Muir J. G. (2014). A diet low in FODMAPs reduces symptoms of irritable bowel syndrome. *Gastroenterology*.

[17] Lopez N. P. y., Torres-Lopez E., Zamarripa-Dorsey F. (2015). Clinical response in Mexican patients with irritable bowel syndrome treated with a low diet low in fermentable carbohydrates (FODMAP). *Revista de Gastroenterología de México*.

[18] Marsh A., Eslick E. M., Eslick G. D. (2016). Does a diet low in FODMAPs reduce symptoms associated with functional gastrointestinal disorders? A comprehensive systematic review and meta-analysis. *European Journal of Nutrition*.

[19] Staudacher H. M., Whelan K. (2017). The low FODMAP diet: recent advances in understanding its mechanisms and efficacy in IBS. *Gut*.

[20] Ong D. K., Mitchell S. B., Barrett J. S. (2010). Manipulation of dietary short chain carbohydrates alters the pattern of gas production and genesis of symptoms in irritable bowel syndrome. *Journal of Gastroenterology and Hepatology*.

[21] Ledochowski M., Widner B., Bair H., Probst T., Fuchs D. (2000). Fructose- and sorbitol-reduced diet improves mood and gastrointestinal disturbances in fructose malabsorbers. *Scandinavian Journal of Gastroenterology*.

[22] Black C. J., Yiannakou Y., Houghton L. A., Ford A. C. (2020). Epidemiological, clinical, and psychological characteristics of individuals with self-reported irritable bowel syndrome based on the Rome IV vs Rome III criteria. *Clinical Gastroenterology and Hepatology*.

[23] Wang J., Yang P., Zhang L., Hou X. (2021). A low-FODMAP diet improves the global symptoms and bowel habits of adult IBS patients: a systematic review and meta-analysis. *Frontiers in Nutrition*.

[24] Moayyedi P., Surette M. G., Kim P. T. (2015). Fecal microbiota transplantation induces remission in patients with active ulcerative colitis in a randomized controlled trial. *Gastroenterology*.

[25] Chen H. T., Huang H. L., Xu H. M. (2020). Fecal microbiota transplantation ameliorates active ulcerative colitis. *Experimental and Therapeutic Medicine*.

[26] Cui B., Li P., Xu L. (2015). Step-up fecal microbiota transplantation strategy: a pilot study for steroid-dependent ulcerative colitis. *Journal of Translational Medicine*.

[27] Drossman D. A., Patrick D. L., Whitehead W. E. (2000). Further validation of the IBS-QOL: a disease-specific quality-of-life questionnaire. *The American Journal of Gastroenterology*.

[28] Patrick D. L., Drossman D. A., Frederick I. O., DiCesare J., Puder K. L. (1998). Quality of life in persons with irritable bowel syndrome: development and validation of a new measure. *Digestive Diseases and Sciences*.

[29] Francis C. Y., Morris J., Whorwell P. J. (1997). The irritable bowel severity scoring system: a simple method of monitoring irritable bowel syndrome and its progress. *Alimentary Pharmacology & Therapeutics*.

[30] Lahtinen P., Jalanka J., Hartikainen A. (2020). Randomised clinical trial: faecal microbiota transplantation versus autologous placebo administered via colonoscopy in irritable bowel syndrome. *Alimentary Pharmacology & Therapeutics*.

[31] Nakada K., Ikeda M., Takahashi M. (2015). Characteristics and clinical relevance of postgastrectomy syndrome assessment scale (PGSAS)-45: newly developed integrated questionnaires for assessment of living status and quality of life in postgastrectomy patients. *Gastric Cancer*.

[32] Maier W., Buller R., Philipp M., Heuser I. (1988). The Hamilton anxiety scale: reliability, validity and sensitivity to change in anxiety and depressive disorders. *Journal of Affective Disorders*.

[33] Miller I. W., Bishop S., Norman W. H., Maddever H. (1985). The modified Hamilton rating scale for depression: reliability and validity. *Psychiatry Research*.

[34] Guo M., Wu F., Hao G. (2017). Bacillus subtilis improves immunity and disease resistance in rabbits. *Frontiers in Immunology*.

[35] Bokulich N. A., Subramanian S., Faith J. J. (2013). Quality-filtering vastly improves diversity estimates from Illumina amplicon sequencing. *Nature Methods*.

[36] Caporaso J. G., Kuczynski J., Stombaugh J. (2010). QIIME allows analysis of high-throughput community sequencing data. *Nature Methods*.

[37] Edgar R. C. (2013). UPARSE: highly accurate OTU sequences from microbial amplicon reads. *Nature Methods*.

[38] Shao H., Zhang C., Xiao N., Tan Z. (2020). Gut microbiota characteristics in mice with antibiotic-associated diarrhea. *BMC Microbiology*.

[39] Cornejo-Granados F., Gallardo-Becerra L., Leonardo-Reza M., Ochoa-Romo J. P., Ochoa-Leyva A. (2018). A meta-analysis reveals the environmental and host factors shaping the structure and function of the shrimp microbiota. *PeerJ*.

[40] Peters B. A., McCullough M. L., Purdue M. P. (2018). Association of coffee and tea intake with the oral microbiome: results from a large cross-sectional study. *Cancer Epidemiology, Biomarkers & Prevention*.

[41] Wang Q., Garrity G. M., Tiedje J. M., Cole J. R. (2007). Naive Bayesian classifier for rapid assignment of rRNA sequences into the new bacterial taxonomy. *Applied and Environmental Microbiology*.

[42] Pruesse E., Quast C., Knittel K. (2007). SILVA: a comprehensive online resource for quality checked and aligned ribosomal RNA sequence data compatible with ARB. *Nucleic Acids Research*.

[43] DeSantis T. Z., Hugenholtz P., Larsen N. (2006). Greengenes, a chimera-checked 16S rRNA gene database and workbench compatible with ARB. *Applied and Environmental Microbiology*.

[44] Segata N., Izard J., Waldron L. (2011). Metagenomic biomarker discovery and explanation. *Genome Biology*.

[45] Huang C., Yi X., Long H. (2020). Disordered cutaneous microbiota in systemic lupus erythematosus. *Journal of Autoimmunity*.

[46] Magge S., Lembo A. (2012). Low-FODMAP diet for treatment of irritable bowel syndrome. *Gastroenterol Hepatol (N Y)*.

[47] Halmos E. P., Christophersen C. T., Bird A. R., Shepherd S. J., Gibson P. R., Muir J. G. (2015). Diets that differ in their FODMAP content alter the colonic luminal microenvironment. *Gut*.

[48] Staudacher H. M., Scholz M., Lomer M. C. (2021). Gut microbiota associations with diet in irritable bowel syndrome and the effect of low FODMAP diet and probiotics. *Clinical Nutrition*.

[49] Rauf A., Khalil A. A., Rahman U. U. (2022). Recent advances in the therapeutic application of short-chain fatty acids (SCFAs): an updated review. *Critical Reviews in Food Science and Nutrition*.

[50] Bourdu S., Dapoigny M., Chapuy E. (2005). Rectal instillation of butyrate provides a novel clinically relevant model of noninflammatory colonic hypersensitivity in rats. *Gastroenterology*.

[51] Tana C., Umesaki Y., Imaoka A., Handa T., Kanazawa M., Fukudo S. (2009). Altered profiles of intestinal microbiota and organic acids may be the origin of symptoms in irritable bowel syndrome. *Neurogastroenterology and Motility*.

[52] Musch M. W., Bookstein C., Xie Y., Sellin J. H., Chang E. B. (2001). SCFA increase intestinal Na absorption by induction of NHE3 in rat colon and human intestinal C2/bbe cells. *American Journal of Physiology. Gastrointestinal and Liver Physiology*.

[53] Suply E., de Vries P., Soret R., Cossais F., Neunlist M. (2012). Butyrate enemas enhance both cholinergic and nitrergic phenotype of myenteric neurons and neuromuscular transmission in newborn rat colon. *American Journal of Physiology. Gastrointestinal and Liver Physiology*.

[54] El-Salhy M., Valeur J., Hausken T., Gunnar Hatlebakk J. (2021). Changes in fecal short-chain fatty acids following fecal microbiota transplantation in patients with irritable bowel syndrome. *Neurogastroenterology and Motility*.

[55] Xu H. M., Huang H. L., Xu J. (2021). Cross-talk between butyric acid and gut microbiota in ulcerative colitis following fecal microbiota transplantation. *Frontiers in Microbiology*.

[56] Sun Q. H., Liu Z. J., Zhang L. (2021). Sex-based differences in fecal short-chain fatty acid and gut microbiota in irritable bowel syndrome patients. *Journal of Digestive Diseases*.

[57] Sperber A. D., Bangdiwala S. I., Drossman D. A. (2021). Worldwide prevalence and burden of functional gastrointestinal disorders, results of Rome Foundation global study. *Gastroenterology*.

[58] Galica A. N., Galica R., Dumitrascu D. L. (2021). Epidemiology of irritable bowel syndrome in Albania. *Journal of Gastrointestinal and Liver Diseases*.

[59] Myneedu K., Deoker A., Schmulson M. J., Bashashati M. (2019). Fecal microbiota transplantation in irritable bowel syndrome: a systematic review and meta-analysis. *United European Gastroenterology Journal*.

