# Supplementary material for: Fecal Microbiota Transplantation Combined with a Low FODMAP Diet for the Treatment of Irritable Bowel Syndrome with Predominant Diarrhea
Source: Oxid Med Cell Longev. 2022 Sep 21;2022:5121496. doi: 10.1155/2022/5121496 (PMC9519354; doi:10.1155/2022/5121496)
Supplement: Supplementary Materials — Table S1: Common low FODMAP and high FODMAP foods. 2, Table S2: The mean relative abundance and P values of each group at all the three levels. 3, Table S3: Relative abundance of genus and Kruskal-Wallis analysis. 4, Table S4: LEfSe analysis between two groups. [file 5121496.f1.docx]

**Supplementary materials**

| **Table S1. Common low FODMAP and high FODMAP foods** | | |
| --- | --- | --- |
|  | Low FODMAP foods  (Recommended) | High FODMAP foods  (Prohibited/Restricted) |
| Fruits | Banana, strawberry, blueberry, cranberry, durian, grape, cantaloupe, melon, Hami melon, kiwi fruit, orange, passion fruit, papaya, orange pomelo, lemon and lime (Note: if it is dry fruit, eat a small amount). | Apple, mango, pear, watermelon, avocado, cherry, peach, plum, prune, persimmon, apricot, blackberry, nectarine, avocado, longan and litchi. |
| Vegetables | Alfalfa, bamboo shoots, bean sprouts, cabbage, carrots, celery, bergamot, ginger, green beans, lettuce, olives, potatoes, pumpkin, sweet red pepper, spinach, zucchini, taro, turnip, sweet potato, corn, cucumber and winter squash. | Asparagus, broccoli, cauliflower, onion, garlic, leek, eggplant, green pepper, mushroom, sweet corn, Dutch bean, green bean, okra, persimmon pepper and tomato. |
| Seasonings | Basil, red pepper, coriander, ginger, lemon grass, mint, parsley, rosemary, thyme, star anise, fennel, soy sauce, mustard and ketchup. | Berry jam, appetizer and pasta sauce. |
| Bean Cereals | Gluten free bread / cereal products, rice, oats, corn porridge, millet, sorghum, quinoa and tapioca powder. | Soybeans, lentils, chickpeas, kidney beans, black beans, broad beans, green beans, peas, red beans, wheat, barley and rye. |
| Milk products | Lactose free milk, oat milk, rice milk, hard cheese and lactose free yogurt. | Milk, goat's milk, sheep's milk, yogurt, soft raw cheese and ice cream. |
| Nut seeds | Almonds (up to 15), pecans (up to 15), chestnuts, hazelnuts, Macadamia nuts, peanuts, pumpkin seeds, sesame seeds, sunflower seeds and walnuts. | Cashew nuts and pistachios. |
| Sweetener | Sweeteners: glucose and sucrose; Honey substitutes: yellow syrup, maple syrup and black cane syrup. | Sweeteners: fructose, fructose syrup, sorbitol, Xylitol (chewing gum), mannitol, maltitol, preserved fruit and honey. |
| Oils | Olive oil | Butter |
| Drinks | Black coffee, herbal tea(weak), orange juice (up to 125ml), mint tea, water, beer (up to 1 cup) and red wine (up to 1 cup). | Coconut water, apple juice, pear juice, mango juice and herbal tea (strong). |
| Meat | Beef, chicken, mutton and pork | All kinds of sausage and processed meat. |

| **Table S2. The mean relative abundance and P values of each group at all the three levels** | | | | | | | |
| --- | --- | --- | --- | --- | --- | --- | --- |
| Level | Taxonomy | Average relative abundance (%) | | | | P value 1 | P value 2 |
|  |  | Pre-FMT | Post-FMT | Pre-FMT + LFD | Post-FMT + LFD |  |  |
| Phylum | *Firmicutes* | 54.8929 | 45.9764 | 48.1309 | 53.6658 | 0.1941 | 0.5885 |
|  | *Proteobacteria* | 18.9736 | 23.4974 | 25.0610 | 19.0853 | 0.1368 | 1.0000 |
|  | *Actinobacteria* | 18.2999 | 16.7613 | 16.4081 | 11.9569 | 0.9138 | 0.3040 |
|  | *Bacteroidetes* | 5.3086 | 8.6626 | 6.7377 | 12.8971 | 0.2793 | 0.1105 |
|  | *Verrucomicrobia* | 0.2638 | 2.3294 | 2.4856 | 0.4667 | 0.0284 | 0.4819 |
|  | *Fusobacteria* | 1.0385 | 0.9907 | 0.2841 | 1.0354 | 0.3169 | 0.9031 |
|  | *Patescibacteria* | 0.5278 | 0.6432 | 0.4512 | 0.5357 | 0.5250 | 0.4989 |
|  | *Euryarchaeota* | 0.0144 | 0.8149 | 0.0163 | 0.0417 | 0.5930 | 0.9513 |
|  | *Cyanobacteria* | 0.4582 | 0.0618 | 0.1187 | 0.0303 | 0.0989 | 0.3169 |
|  | *Tenericutes* | 0.0010 | 0.0063 | 0.0748 | 0.0232 | 0.0610 | 0.7388 |
| Family | *Enterobacteriaceae* | 18.5914 | 21.2444 | 24.0798 | 17.0279 | 0.1677 | 1.0000 |
|  | *Lachnospiraceae* | 14.6858 | 13.8919 | 14.4412 | 12.4778 | 0.9784 | 0.6456 |
|  | *Bifidobacteriaceae* | 14.6282 | 12.7302 | 11.3058 | 7.0394 | 0.6849 | 0.2036 |
|  | *Streptococcaceae* | 9.2693 | 5.2903 | 8.5843 | 5.8463 | 0.0483 | 0.2674 |
|  | *Enterococcaceae* | 5.8225 | 7.8030 | 6.4580 | 7.1107 | 0.1046 | 0.3169 |
|  | *Bacteroidaceae* | 3.8152 | 6.6753 | 4.1352 | 9.1591 | 0.1231 | 0.0483 |
|  | *Ruminococcaceae* | 6.1147 | 4.3158 | 4.7675 | 6.6655 | 0.7868 | 0.2793 |
|  | *Peptostreptococcaceae* | 5.6703 | 3.7229 | 5.2556 | 6.5265 | 0.9784 | 0.9138 |
|  | *Lactobacillaceae* | 6.6726 | 2.2167 | 1.8285 | 7.9655 | 0.7251 | 0.2559 |
|  | *Coriobacteriaceae* | 1.8031 | 2.4511 | 2.5705 | 3.2244 | 0.1046 | 1.0000 |
| Genus | *Escherichia-Shigella* | 14.4961 | 16.9284 | 16.3158 | 14.0421 | 0.0659 | 0.4819 |
|  | *Bifidobacterium* | 14.6176 | 12.7227 | 11.2880 | 7.0316 | 0.6849 | 0.2134 |
|  | *Blautia* | 9.5985 | 9.2158 | 7.7999 | 5.8645 | 0.7455 | 0.2793 |
|  | *Enterococcus* | 5.7785 | 7.7352 | 6.3976 | 7.0428 | 0.1046 | 0.3040 |
|  | *Streptococcus* | 9.2120 | 4.2815 | 8.0935 | 5.0051 | 0.0373 | 0.1298 |
|  | *Bacteroides* | 3.8152 | 6.6753 | 4.1352 | 9.1591 | 0.1231 | 0.0483 |
|  | *Romboutsia* | 5.3947 | 3.4718 | 4.9717 | 6.4298 | 0.9138 | 0.8498 |
|  | *Lactobacillus* | 5.2425 | 1.4302 | 1.6372 | 3.5339 | 0.9569 | 0.6456 |
|  | *Collinsella* | 1.8031 | 2.4511 | 2.5705 | 3.2240 | 0.1046 | 1.0000 |
|  | *Weissella* | 1.0725 | 4.4355 | 1.7662 | 1.2047 | 0.1368 | 0.1298 |
|  | *Subdoligranulum* | 2.7617 | 1.7871 | 2.7019 | 0.9295 | 0.3040 | 0.2914 |
|  | *Pediococcus* | 1.4179 | 0.7769 | 0.1868 | 4.3975 | 0.3302 | 0.9676 |
|  | *Akkermansia* | 0.2613 | 2.3241 | 2.4823 | 0.4636 | 0.0284 | 0.4328 |
|  | *Eubacterium_hallii_group* | 1.0932 | 1.2616 | 1.7657 | 0.8994 | 0.9138 | 0.2674 |
|  | *Erysipelotrichaceae_UCG-003* | 1.6995 | 0.5185 | 1.6295 | 1.0110 | 0.1941 | 0.9784 |
|  | *Dorea* | 0.8864 | 1.0143 | 1.5029 | 1.3438 | 0.3302 | 0.3867 |
|  | *Faecalibacterium* | 1.1011 | 0.8031 | 0.8705 | 1.5787 | 0.7251 | 0.2134 |
|  | *Parabacteroides* | 0.4746 | 1.2929 | 0.2079 | 2.0456 | 0.3438 | 0.0453 |
|  | *Fusobacterium* | 1.0338 | 0.9834 | 0.1150 | 1.0281 | 0.3169 | 0.9138 |
|  | *Actinomyces* | 0.5011 | 0.7060 | 1.1966 | 0.6897 | 0.9353 | 0.7049 |

FMT: fecal microbiota transplantation; LFD: low FODMAP diet; P value 1: Pre-FMT vs. Post-FMT, P value 2: Pre-FMT + LFD vs. Post-FMT + LFD.

| **Table S3. Relative abundance of genus and Kruskal-Wallis analysis** | | | | | |
| --- | --- | --- | --- | --- | --- |
| Genus | Pre-FMT group | Post-FMT group | Pre-FMT + LFD group | Post-FMT + LFD group | P-value |
| *Bifidobacterium* | 14.6176 | 12.7227 | 11.2880 | 7.0316 | 0.0484 |
| *Bacteroides* | 3.8152 | 6.6753 | 4.1352 | 9.1591 | 0.0498 |
| *Akkermansia* | 0.2613 | 2.3241 | 2.4823 | 0.4636 | 0.0390 |
| *Alistipes* | 0.16146 | 0.2418 | 0.1168 | 0.5173 | 0.0441 |
| *Eubacterium_eligens_group* | 0.0381 | 0.1079 | 0.0337 | 0.5747 | 0.0277 |
| *Ruminococcaceae_UCG-002* | 0.0550 | 0.0478 | 0.0571 | 0.2093 | 0.0429 |

FMT: fecal microbiota transplantation; LFD: low FODMAP diet.

| **Table S4. LEfSe analysis between two groups** | | | | |
| --- | --- | --- | --- | --- |
| Taxonomy | Level | Group | LDA score | P value |
| *Akkermansiaceae* | Family | Post-FMT | 3.9772 | 0.0068 |
| *Variovorax_paradoxus* | Species | Post-FMT | 3.4725 | 0.0377 |
| *Leuconostocaceae* | Family | Post-FMT | 4.2969 | 0.0425 |
| *Prevotellaceae_UCG_001* | Genus | Post-FMT | 2.9311 | 0.0274 |
| *Bifidobacteriaceae* | Family | Post-FMT | 4.4734 | 0.0186 |
| *Bifidobacterium* | Genus | Post-FMT | 4.4734 | 0.0186 |
| *Verrucomicrobia* | Phylum | Post-FMT | 3.9775 | 0.0068 |
| *Allorhizobium_Neorhizobium_Pararhizobium_Rhizobium* | Genus | Post-FMT | 3.4451 | 0.0377 |
| *Bifidobacteriales* | Order | Post-FMT | 4.4734 | 0.0186 |
| *Actinobacteria* | Class | Post-FMT | 4.4742 | 0.0215 |
| *Lactobacillus_mucosae* | Species | Post-FMT | 2.5439 | 0.0280 |
| *Sphingomonadaceae* | Family | Post-FMT | 3.2044 | 0.0262 |
| *Terrimonas* | Genus | Post-FMT | 3.6652 | 0.0377 |
| *Sphingomonadales* | Order | Post-FMT | 3.1075 | 0.0262 |
| *Weissella_paramesenteroides* | Species | Post-FMT | 4.2864 | 0.0425 |
| *Weissella* | Genus | Post-FMT | 4.2907 | 0.0373 |
| *Akkermansia* | Genus | Post-FMT | 3.9772 | 0.0068 |
| *Verrucomicrobiae* | Class | Post-FMT | 3.9775 | 0.0068 |
| *Verrucomicrobiales* | Order | Post-FMT | 3.9773 | 0.0068 |
| *Variovorax* | Genus | Post-FMT | 3.5474 | 0.0377 |
| *Sphingomonas* | Genus | Post-FMT | 3.0591 | 0.0324 |
| *Acidobacteriaceae_Subgroup_1* | Family | Post-FMT | 3.1517 | 0.0454 |
| *Lachnospira* | Genus | Post-FMT + LFD | 2.5386 | 0.0424 |
| *Christensenellaceae* | Family | Post-FMT + LFD | 2.9843 | 0.0119 |
| *Parabacteroides_goldsteinii* | Species | Post-FMT + LFD | 3.1771 | 0.0372 |
| *Desulfovibrio_desulfuricans_subsp_desulfuricans* | Species | Post-FMT + LFD | 2.4151 | 0.0229 |
| *Barnesiellaceae* | Family | Post-FMT + LFD | 2.6042 | 0.0372 |
| *Pseudomonas_veronii* | Species | Post-FMT + LFD | 3.4164 | 0.0081 |
| *Ruminococcaceae_UCG_002* | Genus | Post-FMT + LFD | 2.8038 | 0.0200 |
| *Caproiciproducens* | Genus | Post-FMT + LFD | 2.9018 | 0.0419 |
| *Pediococcus* | Genus | Post-FMT + LFD | 4.1446 | 0.0398 |
| *Bacteroides_sp* | Species | Post-FMT + LFD | 2.8048 | 0.0004 |
| *Christensenellaceae_R_7_group* | Genus | Post-FMT + LFD | 2.9484 | 0.0483 |
| *Eubacterium_eligens_group* | Genus | Post-FMT + LFD | 3.1982 | 0.0040 |
| *Pediococcus_pentosaceus* | Species | Post-FMT + LFD | 4.1424 | 0.0398 |
| *Anaerofustis* | Genus | Post-FMT + LFD | 2.9035 | 0.0212 |
| *Acidaminococcus_fermentans_DSM_20731* | Species | Post-FMT + LFD | 2.7634 | 0.0090 |
| *Eubacterium_oxidoreducens_group* | Genus | Post-FMT + LFD | 3.1282 | 0.0155 |
| *Anaerofustis_stercorihominis_DSM_17244* | Species | Post-FMT + LFD | 2.8997 | 0.0212 |
| *CAG_56* | Genus | Post-FMT + LFD | 2.5386 | 0.0453 |
| *Paraprevotella* | Genus | Post-FMT + LFD | 2.9595 | 0.0304 |

FMT: fecal microbiota transplantation; LFD: low FODMAP diet.
